# Pre-Digested Foods

**Published:** 1887-06

**Authors:** 


### PRE-DIGESTED FOODS.

The prejudice against prepared foods, is fast wearing away. The medical profession already regards them as useful, almost invaluable for infants and invalids, and recommends their use. The adoption of artificial digestive agents in cases of weakness and failure of the digestive powers, is also resorted to. This incapacity is more common than one would think, coming from mental worry, exhaustion, intemperance, the opium habit, disease, accident, negligence, etc., and nervous people are proverbially dyspeptic.

The American habit of fast eating is very injurious to good digestion. The body demands for the muscles—food rich in nitrogen; for the maintenance of animal heat—carbonaceous food; for the brain and nerves—phosphates. We find albumen in the muscles and flesh, thus proving its necessity for the building up of tissue. But when the Liver is deranged, the digestion of albuminoids cannot properly be performed, hence only in a pre-digestive form can it be assimilated by the system.

Albumen is found in milk and in smaller proportion in cereals as the gluten of wheat. Phosphate of lime is required for the bones, and is furnished by cereals and milk. Milk Sugar is desirable for old people and children.

"Baby Foods" generally lack fat ; fat is essential to healthy tissue, and therefore foods containing milk are the most complete, as a certain amount of fat is present. The principle which should underlie all baby foods, is the conversion of insoluble starch into soluble matters, to prevent its irritant presence setting up diarrhœa for its removal. When cereals are cooked by high steam heat, the starch transformation into soluble dextrine is more complete The digestive organs become enfeebled by habits of civilization, hence the predigested starch must come more and more to the front. Babies have their choice of food just as much as their elders, and they show it by the rejection of one food and delight in another. They also show ability to digest and assimilate easily and thoroughly the food. But because one kind or form of prepared food is distasteful and disagreeable, it does not follow that all are so. Wells, Richardson & Co., of Burlington, Vt., manufacture "Lactated Food," which is a restorative and constructive in various conditions of the system. Their preparation is meeting with great success in the diet of invalids and children, and is received with approval by food experts, at home and abroad.

Analysis shows its component parts more nearly similar to mother's milk than is cow's milk. Its nutritive elements are derived from the three great cereals, Wheat, Barley and Oats. From Wheat is taken the pure gluten ; from the Barley, all the soluble albuminoid and extractive matter resulting from the most careful malting ; and from the Oat, the strengthening properties for which it is so well-known. From the fact that it is partially digested in process of preparation, it is assimilated by the feeblest stomach, and no undigested particles pass into the bowels to irritate, and thus cause troublesome and dangerous bowel troubles.

Its basis is Milk Sugar, which never causes acetic fermentation. The gluten flour is partially torrefied and every particle is subjected to the action of the malto-diastase, thus transforming the starch into soluble carbo-hydrates ; so that where by reason of weakening the natural forces, and impairing the digestive functions, the conversion of starch is so slight, that the stomach is hampered and strained, nutrition may be kept up by the use of prepared foods. And when, in the case of infants deprived of mother's nursing, cow's milk disagrees, and a wet nurse renders its chances of life precarious, Lactated Food is the sole reliance and support.

